# Dreaming in Adolescents During the COVID-19 Health Crisis: Survey Among a Sample of European School Students

**DOI:** 10.3389/fpsyg.2021.652627

**Published:** 2021-04-20

**Authors:** Ana Guerrero-Gomez, Isabel Nöthen-Garunja, Michael Schredl, Annelore Homberg, Maria Vulcan, Asja Brusić, Caterina Bonizzi, Cecilia Iannaco

**Affiliations:** ^1^European Network for Psychodynamic Psychiatry, Rome, Italy; ^2^Sleep Laboratory, Central Institute of Mental Health, Medical Faculty, Mannheim/Heidelberg University, Mannheim, Germany; ^3^Timișoara 2021 – European Capital of Culture Association, Timișoara, Romania; ^4^Rijeka 2020 – European Capital of Culture, Croatian Cultural Centre, Rijeka, Croatia

**Keywords:** school students, COVID-19 health crisis, lockdown, dreaming, nightmares, emotional distress, adolescence

## Abstract

According to the continuity hypothesis of dreaming and contemporary psychodynamic approaches, dreams reflect waking life. The aim of the present study was to explore the relationship between the COVID-19 pandemic and dreaming in adolescents. A cross-sectional survey was conducted in Italy, Romania and Croatia involving 2,105 secondary school students (69% girls, mean age 15.6 ± 2.1 years; 31% boys, mean age 15.1 ± 2.2 years; mean age of whole sample 15.4 ± 2.1 years). No substantial differences between countries were found. Thirty-one percent of the participants reported heightened dream recall, 18% noticed an increase in nightmares during the lockdown, and 15% of the provided dreams (*n* = 498) included pandemic-related content. The results indicate that subjective emotional reactions to lockdown had a significantly higher correlation to dreaming than objective distress (i.e., illness or death of a close one because of COVID-19). These findings suggest that attention to dreams should be included in preventive programs for adolescents with pandemic-related stress.

## Introduction

Dreams reflect waking life, according to the continuity hypothesis of dreaming (Schredl, 2003) and psychodynamic approaches (Fagioli, 1972; Iannaco et al., 2015), and there is evidence that emotionally significant waking life experiences are integrated in dreams (Strauch and Meier, 1996; Domhoff, 2018; Schredl, 2018). Given these theoretical frameworks and findings, it is likely to expect that the coronavirus disease 2019 outbreak, along with the pandemic-related restrictions, might have affected dreaming.

Recent studies showed indeed that dreaming in adults has undergone significant changes during the COVID-19 pandemic (Barrett, 2020; Mota et al., 2020; Gorgoni et al., 2021; Wang et al., 2021). Regarding dream recall in particular, several studies (Bottary et al., 2020; Pappa et al., 2020; Gorgoni et al., 2021) indicated a self-reported increase in dream recall due to the pandemic; this could be explained by the longer sleep duration during home confinement (Martínez-Lezaun et al., 2020) or by changes in sleep pattern***s*** due to home working (Altena et al., 2020; Cellini et al., 2020).

Moreover, a shift toward more negative dreams was found, which was directly related to the subjective stress in waking life, for example, social distancing affected mental health (Barrett, 2020; Iorio et al., 2020; Mota et al., 2020; Schredl and Bulkeley, 2020). In a similar way, the frequency of nightmares increased during the pandemic in both clinical (Gupta, 2020; Sierro et al., 2020) and normative samples of adults (Musse et al., 2020; Pérez-Carbonell et al., 2020; Scarpelli et al., 2021).

As to dreams with direct references to the pandemic, studies reported different frequencies of COVID-19-related dream content, varying from 8.2% (Schredl and Bulkeley, 2020) to 55% (Pesonen et al., 2020). Most dreams with pandemic-related content reflected the participants' fears of contracting the virus or close persons becoming ill or dying (Iorio et al., 2020; MacKay and DeCicco, 2020).

All reported studies focus on adults; there is a lack of empirical studies on dreaming in adolescents during the COVID-19 crisis. But the pandemic also changed adolescents' daily lives in a consistent way due to home confinement, school closures and suspension of sport, cultural, and leisure activities. One of the few studies on adolescents' dreaming during the pandemic carried out so far (Parrello et al., 2021) examined adolescents who had had close persons infected by or who had died of COVID-19; these subjects reported a more negative emotional tone to their dreams, more nightmares and more COVID-19-related dreams compared to adolescents without these dramatic experiences, similar to the findings in adults.

As the realization of peer relationships is an essential condition for adolescents' mental health, adolescents might have been affected particularly by pandemic-induced isolation (Commodari and La Rosa, 2020; Loades et al., 2020; UNESCO, 2020; Xiang et al., 2020). In Europe, the lockdown measures and thus the degree of social isolation varied for each country. In most countries, however, teaching at school was suspended and distance learning in home confinement was introduced.

The objective of the present study was to examine the effects of the lockdown caused by the COVID-19 pandemic on adolescents' dreaming in three different European countries. We expected an increase in dream recall and more nightmares and COVID-19-related dream content, especially in those adolescents who had experienced a traumatic impact of COVID-19 in their family (infected members or deaths).

## Materials and Methods

### Participants

The present study consisted of an international cross-sectional survey among adolescents from three different European countries (Italy, Romania, and Croatia). In total, 2,105 adolescents completed the questionnaire: 1,446 girls (68.7%, mean age 15.6 ± 2.1 years) and 659 boys (31.3%, mean age 15.1 ± 2.2 years). Mean age of whole sample was 15.4 ± 2.1 years; the participants' ages ranged from 11 to 20 years, with students of 14–20 years of age being the most represented group (11–13 years: 19.7%; 14–16 years: 44.5%; 17–20 years: 35.8%). The majority were from Italy (44.1%) and Romania (47.7%), with only 8.2% from Croatia. Most of the respondents (87.3%) lived in densely populated areas.

### Measures

Adolescents were interviewed using an anonymous online self-report questionnaire developed by the Adolescent Day Hospital, Sapienza University Rome. The questionnaire “My life during the lockdown” is composed of 73 mostly close-ended questions. It was translated from Italian into Romanian, Croatian, and English using a back-translation procedure.

#### Outcomes

The outcomes according to the objectives of this study were: dream recall increase; nightmare increase; report of an extraordinary dream; and pandemic-related dream. All outcomes referred to the period of national lockdown.

Dream recall increase was assessed by asking the participants to complete the sentence “During this time you felt like you were dreaming…” with the following three options: “more than before,” “same as before,” or “less than before.”

To assess nightmare increase, participants responded to the question “Did you have more nightmares than before?” with either “yes” or “no.” There was no precise definition of the term “nightmare” given to the participants.

With regard to “report of an extraordinary dream,” the questionnaire asked for a short written report of a dream: “If you don't mind, please tell us briefly about a dream that struck you during this time.” This item was coded “yes” if the dream report was given and “no” if not.

For each reported dream, “pandemic-related dream” was coded by two independent raters who each had a university degree in psychology. Instructions were given to the raters that pandemic-related content should be scored if the dreams overtly referred to a COVID-19-related topic, with issues and scenarios such as: coronavirus, COVID-19, pandemic, COVID infection, swab, anti-COVID vaccination/medication, hospitalization because of COVID, lockdown, social distancing, domestic isolation, face mask, and other COVID precautions, home schooling, home working, etc.

#### Predictors

Predictors considered in this study were the socio-demographic variables of age (11–20 years), gender (female, male), and country (Italy, Romania, Croatia), as well as a series of other variables dealing with the students' objective and subjective pandemic-related distress, their lockdown management and their emotional condition during domestic isolation, as described below.

The items *Loved One with COVID-19* (“Has somebody dear to you contracted SARS-CoV2?”) and *Loved One Died of COVID-19* (“Have you experienced the loss of a loved one because of SARS-CoV2?”) explored the students' objective stress caused by the pandemic. Two other items dealt with the students' subjective fears of contagion: *Fear of Getting COVID-19* (“Were you afraid of contracting SARS-CoV2?”) and *Fear of Loved Ones Getting COVID-19* (“Were you afraid that somebody close to you might contract SARS-CoV2?”). All these questions had binary answer options.

The items *Suffering from Restrictions* (“How difficult was/is it for you to respect the government-imposed restrictions?”) and *Worries About Another Lockdown* (“How worried are you to be put in the same situation again?”) were answered on a five-point scale: not at all (1), a little (2), quite (3), very (4), and extremely (5).

Overall performance during domestic isolation was explored by the items *Difficulties in Coping with Lockdown* (“How do you think you managed in this period?”) and *Proud about Own Behavior* (“Are you proud of doing something positive for your fellow citizens by obeying the restrictions?”), with binary answer options. The students were also asked about their *Reaction to School Interruption* (“How do you feel about the interruption of your usual school life?”), with the answer options of positive, indifferent, and negative.

Emotional reaction to lockdown was explored by the item *Mood Affected* (“Do you think that the lockdown affected your mood?”). This question could be answered with: yes, in a positive way; no; and yes, in a negative way. A range of emotional reactions was displayed in the items *Experiencing Discomfort/Sadness* (“During domestic isolation, did you ever experience feelings of great discomfort or sadness?”), *Experiencing Anger/Restlessness* and *Experiencing Emptiness/Persistent Boredom*. These items were answered on a five-point scale never (1), rarely (2), sometimes (3), frequently (4), and always (5).

Another group of items explored the students' relationships. The item *Changes Relationship with Parents* was explored by two questions: “Has your relationship with your friends changed during this quarantine?” (answer options: yes or no) and “If yes: has it changed in a positive or in a negative way?” (answer options: positively or negatively). For analysis, the answers of both questions were aggregated into positive change, negative change, and no change. The same type of aggregation was applied to the item *Changes Relationship with Friends* which was also assessed by two questions: “Has your relationship with your friends changed during this quarantine?” (answer options: yes or no) and “If yes: has it changed in a positive or in a negative way?” (answer options: positively or negatively). The question *Missing Social Contacts* (“Did you miss your social contacts and relationships?”) should be answered on a five-point scale: hugely (1), very much (2), quite a bit (3), a bit (4). and not at all (5). For analysis, answers (1) to (4) were aggregated into “yes” and answer (5) remained “no.” The same type of aggregation was applied for the item *Creative Time* (“Do you think that you spent this period of time in a productive and creative way?”).

### Recruitment and Procedure

**Figure 1:**
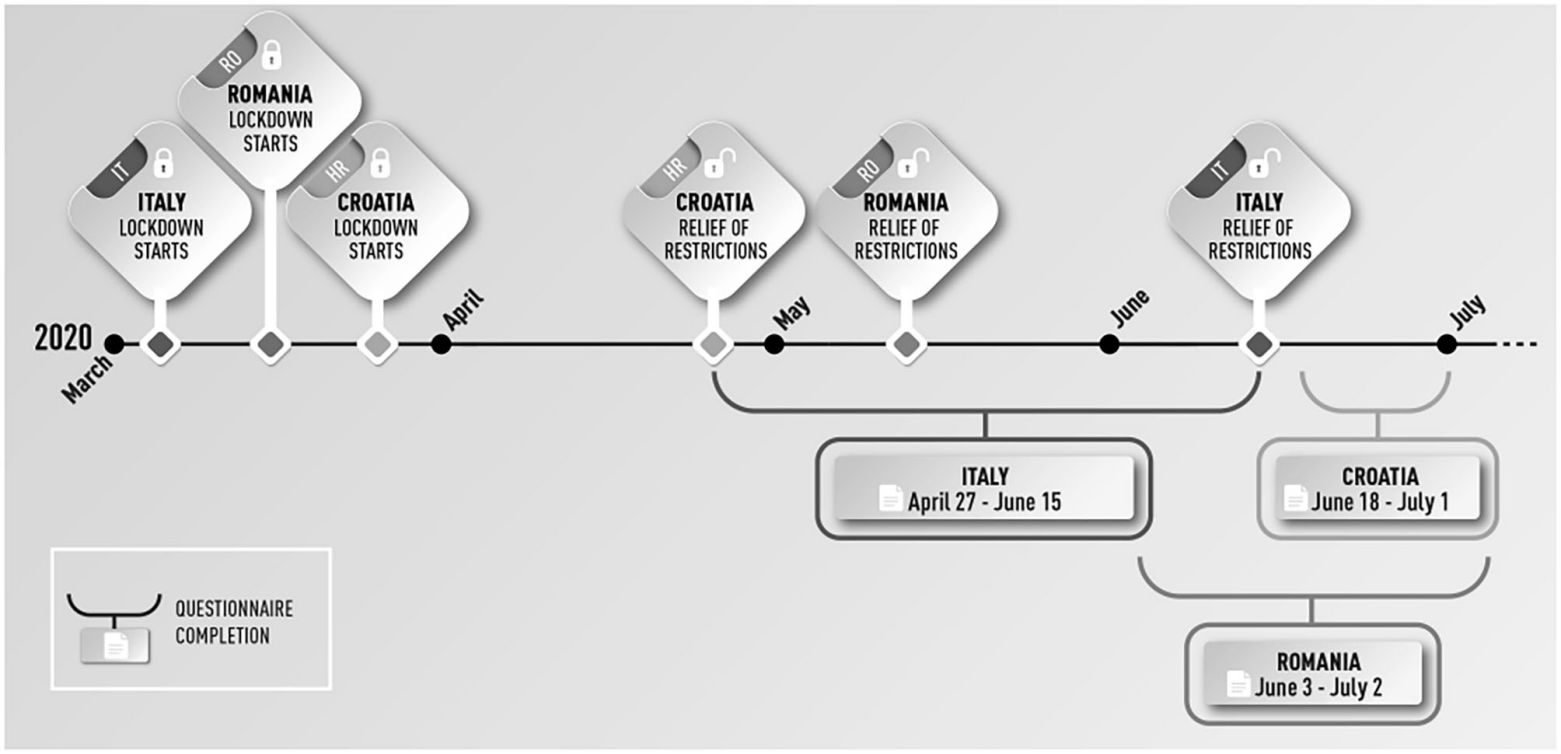
Timeframe of lockdown restrictions and questionnaire completion in Italy, Romania, and Croatia.

During and immediately after the COVID-19 lockdown measures (Figure 1) data collection was coordinated by Netforpp Europa in Italy, by the association *Timișoara 2021* – *European Capital of Culture Association* in Romania and by *Hrvatski kulturni dom na Sušaku* (Croatian Cultural Center Rijeka) in Croatia.

Italian Schools were based mainly in Rome and Florence. The Croatian coordinator collaborated with the Departments of Education and Schooling of the City of Rijeka and Primorsko-goranska County, which distributed the questionnaire among schools in Rijeka and its region. In order to obtain a nationwide coverage and adequate distribution of age groups, the coordinator in Romania collaborated with the “Europe Direct” network of information centers in Arad and Bucharest, the Timis Sibiu County School Inspectorates and several media partners. About half (*n* = 508) of the total number of 1,004 responses in Romania were collected in the “Elena Ghiba Birta” National College in Arad. The other half of responses came from schools in Timișoara, Sibiu, and Resifa.

Every school was contacted 2 weeks before the survey started and the study protocol was outlined in detail to head and class teachers.

Each participant of the questionnaire remained anonymous and respondents' IP addresses could not be disclosed. Participation was entirely voluntary and without any compensation. Participants over the age of 18 years gave their voluntary informed consent to participate in the research before taking part in the study. For students under 18 years of age, Italian participating schools entered the questionnaire in the school's electronic parental platform, together with a cover letter in which all the information on the study protocol and the survey's objectives were given. Only after parental acknowledgment and consent could students under the age of 18 years fill out the questionnaire. The survey thus followed privacy guidelines released by the Italian Ministry of Education, University and Research (MIUR, 2020) and was conducted in accordance with the Declaration of Helsinki.

### Statistical Analysis

We analyzed the data using SAS/STAT® software (SAS 9.4, SAS Institute Inc., Cary, NC, USA) and computed logistic and ordinal regression analyses for all four outcomes and the predictors of age, gender, and country (**Table 2**). Because of their impact on various outcomes, these predictors were controlled for in the subsequent regression analyses. In a first step, we computed regression analysis for all waking life variables that are associated with possible distress due to the pandemic with increase in nightmares in order to determine the strength of the associations. As waking life stressors were inter-correlated, the regression analyses with simultaneous entered variables were computed to control for these inter-correlations. Logistic regressions with the outcome “nightmare increase” and 22 predictors were computed, while ordinal and logistic regression on “dream recall increase,” “nightmare increase,” and “report of an extraordinary dream” were performed with 16 predictors and a logistic regression on “pandemic-related dream” was performed with 13 predictors. The outcome “pandemic-related dream” had to be computed separately because of varying sample size. Effect size *d* was computed using the online tool Psychometrica (Lenhard and Lenhard, 2016). We did not specify a particular critical *p*-value but presented the exact *p*-value. Given the large sample size and the number of statistical tests, we focused mainly on findings with *p* < 0.001 and substantial effect sizes.

## Results

### Reported Effects of the Pandemic

Descriptive statistics of the sample (*n* = 2,105) regarding the binary and multi-stage variables used for analyses can be found in Table 1. Numbers of subjects reporting fear of being infected by COVID-19 (46.9%) and fear of loved ones getting infected (76.2%) were relatively high. Likewise, difficulties in coping with lockdown (85.1%) and missing social contacts (81.1%) affected a large number of students. Most reactions to school interruption were negative (46.8%), with 20.9% feeling the interruption was positive and 32.3% being indifferent. Regarding *Mood affected*, almost half of the sample (46.1%) reported a negatively changed mood during lockdown, 16.9% indicated positive mood changes and 37% said their mood had not been affected. The majority of the students (76%) reported positive changes in the relationships with their parents, 17.6% reported negative changes and only a few students (6.4%) indicated that the relationship had not changed at all. Similarly, for relationships with friends the majority of respondents (61.4%) reported positive changes, nearly one-third (31.7%) reported that their relationships with friends changed negatively and 6.9% said there was no change at all.

**Table 1:** Descriptive statistics of the sample characteristics (*n* = 2,105).

	**% / M ± SD**
Nightmare increase (yes)	18.19
Reporting an extraordinary dream (yes)	23.75
Loved one with COVID-19 (yes)	6.70
Loved one died of COVID-19 (yes)	1.52
Fear of getting COVID-19 (yes)	46.89
Fear of loved ones getting COVID-19 (yes)	76.25
Worries about another lockdown	2.96 ± 1.20
Suffering from restrictions	2.25 ± 1.01
Difficulties in coping with lockdown (yes)	85.08
Proud about own behavior (yes)	86.13
Experiencing discomfort/sadness	2.84 ± 1.13
Experiencing anger/restlessness	2.66 ± 1.13
Experiencing emptiness/persistent boredom (*n* = 2,099)	3.14 ± 1.18
Missing social contacts (yes)	81.19
Creative time (yes)	62.23

*For all binary variables, percentages were reported; for all multi-stage variables, means, and standard deviations (M ± SD) were reported; where n differed from 2,105, this was reported*.

### Dream Recall Increase

The dream recall increase experienced during the COVID-19 pandemic is described in Table 2. About 31% reported an increase in dream recall whereas about 12% reported a decrease. For most participants (57%), there was no observed change in dream recall. Regression analysis indicated that older participants and girls were more likely to report a dream recall increase (Table 3); this was confirmed also by ordinal and logistic regression (see **Table 5**). In this regression, *Experiencing Discomfort/Sadness* also had an impact on dream recall; those who reported feelings of discomfort and sadness were also more likely to report a dream recall increase (see **Table 5**). Living in Italy, Croatia, or Romania was not associated with this variable (Table 3).

**Table 2:** Dream recall increase due to the pandemic (*n* = 2,105).

	**Frequency**	**Percentage**
Dream recall increase	646	30.69
No change	1,208	57.39
Dream recall decrease	251	11.92

**Table 3:** Logistic and ordinal regression analyses for Dream recall increase, Nightmare increase due to the COVID-19 pandemic and Reporting an extraordinary dream that struck the participant during the pandemic.

	**Dream recall increase**				**Nightmare increase**				**Reporting an extraordinary dream**			
	***SE***	***X^2^***	***p***	***d***	***SE***	***X^2^***	***P***	***d***	***SE***	***X^2^***	***p***	***d***
Age	0.0971	16.4	<0.0001	0.177	0.1443	18.3	<0.0001	0.187	−0.0710	6.1	0.0134	0.108
Gender	0.1023	17.9	<0.0001	0.185	0.2342	37.6	<0.0001	0.270	0.1098	13.2	0.0003	0.159
Italy vs. Croatia	−0.0181	0.5	0.4670	0.031	−0.0826	4.9	0.0262	0.097	−0.0941	8.1	0.0045	0.124
Italy vs. Romania	−0.0046	0.0	0.8534	0.008	−0.1040	9.9	0.0017	0.138	−0.0637	4.6	0.0311	0.094

### Nightmare Increase

Eighteen percent of the participants reported an increase in nightmares. Regression analysis indicated that older participants were more likely to report an increase in nightmares during the lockdown (Table 3). In addition, gender was significantly associated with this outcome (Table 3); 22.2% of girls and 9.4% of boys indicated that they had more nightmares than before. There were small but significant differences between countries, with Italian participants (22.4%) more likely to report “nightmare increase” during lockdown than Croatian (12.7%) and Romanian (15.2%).

Logistic regressions indicate that variables linked to subjective distress were positively associated with “nightmare increase” (Table 4): for example, reporting more *Suffering from Restrictions, Worries about Another Lockdown, Fear of Getting COVID-19* and *Difficulties in Coping with Lockdown* (Table 4). Also, emotional reactions, assessed by the variables *Mood Affected, Experiencing Discomfort/Sadness, Experiencing Anger/Restlessness*, and *Experiencing Emptiness/Persistent Boredom*, were significantly associated with this outcome, with medium effect sizes (Table 4).

**Table 4:** Logistic regressions of Nightmare increase due to the COVID-19 pandemic (*n* = 2,105).

	***SE***	***X^2^***	***p***	***d***
Suffering from restrictions	0.1602	25.5	<0.0001	0.222
Proud about own behavior	−0.0196	0.3	0.5555	0.024
Worries about another lockdown	0.2264	40.2	<0.0001	0.279
Fear of getting COVID-19	0.1079	11.1	0.0009	0.146
Fear of loved ones getting COVID-19	0.0931	6.1	0.0137	0.108
Loved one with COVID-19	0.0694	6.4	0.0114	0.110
Loved one died of COVID-19	0.0497	3.8	0.0514	0.085
Mood affected	−0.1575	21.0	<0.0001	0.201
Experiencing discomfort/sadness	0.4592	129.5	<0.0001	0.512
Experiencing anger/restlessness	0.4040	119.5	<0.0001	0.491
Experiencing emptiness/persistent boredom	0.3056	68.0	<0.0001	0.365
Reaction to school interruption	−0.0295	0.8	0.3626	0.039
Difficulties in coping with lockdown	0.1336	10.3	0.0013	0.140
Changes relationship parents	−0.1523	22.8	<0.0001	0.209
Changes relationship friends	−0.0779	5.3	0.0215	0.101
Missing social contacts	−0.1607	21.8	<0.0001	0.205
Creative time	−0.0776	6.1	0.0139	0.108

*Each variable was tested separately, with age, gender, and country as additional predictors (not depicted)*.

Variables connected to objective distress, such as *Loved One with COVID-19* and *Loved One Died of COVID-19*, also showed a significant association to “nightmare increase,” even though effect sizes were small (Table 4).

Negative changes in relationships with parents and friends were predictive of an increase in nightmares. Students who reported not missing their social contacts were significantly more likely to report an increase in nightmares than those who reported missing them (Table 4).

Ordinal and logistic regression found that all significant predictors were specific to gender and type of emotion, with more girls reporting an increase in nightmares and the emotion variables *Experiencing Discomfort/Sadness* and *Experiencing Anger/Restlessness*; students who reported feeling sad or angry more often during domestic isolation indicated that they also had more nightmares (Table 5).

**Table 5:** Ordinal and logistic regression for Dream recall increase, Nightmare increase due to COVID-19 pandemic, and Reporting an extraordinary dream that struck the participant during the pandemic (*n* = 2,099).

	**Dream recall increase**				**Nightmare increase**				**Reporting an extraordinary dream**			
	***SE***	***X^2^***	***p***	***d***	***SE***	***X^2^***	***P***	***d***	***SE***	***X^2^***	***p***	***d***
Age	0.0645	6.6	0.0100	0.112	0.0414	1.3	0.2633	0.050	−0.1250	16.4	<0.0001	0.178
Gender	0.0724	8.0	0.0048	0.130	0.1173	8.1	0.0044	0.125	0.0556	3.0	0.0853	0.076
Italy vs. Croatia	0.0007	0.0	0.9791	0.000	0.0145	0.1	0.7219	0.014	−0.0656	3.6	0.0595	0.083
Italy vs. Romania	0.0268	0.9	0.3329	0.041	−0.0256	0.4	0.5113	0.028	−0.0512	2.3	0.1273	0.066
Suffering from restrictions	−0.0420	2.3	0.3329	0.066	0.0233	0.4	0.5244	0.028	−0.0444	1.8	0.1853	0.059
Worries about another lockdown	0.0335	1.2	0.2761	0.048	0.0663	2.5	0.1125	0.069	−0.0508	1.9	0.1723	0.060
Fear of getting COVID-19	−0.0225	0.7	0.3980	0.037	0.0717	3.8	0.0526	0.085	0.0508	2.5	0.1158	0.069
Fear of loved ones getting COVID-19	0.0456	2.8	0.0968	0.073	0.0191	0.2	0.6618	0.020	−0.0049	0.0	0.8872	0.000
Loved one with COVID-19	−0.0143	0.3	0.5688	0.024	0.0304	0.9	0.3305	0.041	0.0497	3.1	0.0778	0.077
Loved one died of COVID-19	0.0248	1.0	0.3192	0.044	0.0144	0.2	0.6269	0.020	−0.0181	0.4	0.5350	0.028
Experiencing discomfort/sadness	0.0937	6.5	0.0109	0.111	0.2558	22.8	<0.0001	0.209	0.1460	10.7	0.0011	0.143
Experiencing anger/restlessness	0.0499	2.4	0.6592	0.068	0.2176	22.0	<0.0001	0.206	0.1295	11.0	0.0009	0.145
Experiencing emptiness/persistent boredom	0.0134	0.2	0.6592	0.020	0.0710	2.5	0.1149	0.069	0.0469	1.6	0.2090	0.055
Reaction to school interruption	0.0459	3.3	0.0677	0.080	0.0425	1.4	0.2301	0.052	0.0222	0.5	0.4670	0.031
Difficulties in coping with lockdown	−0.0485	3.5	0.0626	0.082	0.0188	0.2	0.6812	0.020	−0.0219	0.4	0.5073	0.028
Creative time	0.0060	0.1	0.8092	0.014	0.0248	0.5	0.4785	0.031	0.0690	5.0	0.0279	0.098

*All variables were entered simultaneously*.

### Report of an Extraordinary Dream

23.7% of the participants reported a dream that had struck them as extraordinary during lockdown; these dreams were written down more often by girls than by boys. This difference was statistically significant (Table 3). Writing down an extraordinary dream was more likely in the Italian sample (26.9%) than in the other two samples from Romania (22%) and Croatia (16.8%; Table 3).

Logistic regression shows that age (younger teenagers, significantly, produced more written dreams than older ones), experiencing discomfort/sadness and anger/restlessness more often and spending creative time during home confinement increased the probability of writing down an extraordinary dream (Table 5).

### Pandemic-Related Dream

Of the reported dreams (*n* = 498), 14.2% referred directly to a COVID-19-related topic; for example, participants wrote the following in their dream reports: “that they had found the vaccination,” “a bus full with people despite the rules against infection,” “that my mum died of coronavirus,” “Phase 2 was over and we could get back to normal and I resumed dance classes,” “a positive test result for COVID.”

In the logistic regression, age was significantly linked to pandemic-related topics in the reported dream (Table 6). The younger the teenagers were, the more often their dreams dealt overtly with COVID-19. Likewise, the dreams of girls more often showed COVID-19-related dream content. *Worries about another lockdown* and *Creative time* were also significantly related to reporting a pandemic-related dream. No other variable showed a significant relationship (Table 6).

**Table 6:** Logistic regression for Reporting a pandemic-related dream (subsample of participants reporting a dream, *n* = 498).

	***SE***	***X^2^***	***p***	***d***
Age	−0.2796	11.1	0.0008	0.146
Gender	−0.1640	4.2	0.0396	0.090
Worries about another lockdown	0.0323	13.3	0.0003	0.160
Fear of getting COVID-19	−0.0036	0.0	0.9655	0.000
Fear of loved ones getting COVID-19	0.0442	0.2	0.6202	0.020
Loved one with COVID-19	−0.0141	0.0	0.8705	0.000
Loved one died of COVID-19	−0.0329	0.2	0.6977	0.020
Experiencing discomfort/sadness	0.1934	3.0	0.0851	0.076
Experiencing anger/restlessness	−0.0088	0.0	0.9282	0.000
Experiencing emptiness/persistent boredom	0.0315	0.1	0.7457	0.014
Reaction to school interruption	0.0123	0.0	0.8750	0.000
Difficulties in coping with lockdown	−0.1247	2.5	0.1124	0.070
Creative time	0.2623	8.8	0.0030	0.130

## Discussion

Overall, the findings indicate that there is a consistent correlation between the students' emotional stress related to the pandemic and dreaming (i.e., heightened dream recall in general, nightmare increase, and reports of a dream that struck them as extraordinary). Based on the findings that indicated a strong effect of waking life on dreaming (Domhoff, 2018; Schredl, 2018), one might hypothesize that stressors in waking life which are related to the pandemic influence or even determine dreaming. On the other hand, distressing dreams can have a negative effect on waking life emotions. Therefore, simple causality cannot be established; the negative effects of the pandemic on waking life and dreaming processes might interact. In our findings, there were small differences between the three countries, with stronger correlation between emotional stress factors in waking life and dreaming in Italian adolescents; this could be explained, in our opinion, by the critical condition of Italy during the first months of the pandemic which might have induced stronger emotional responses in the Italian adolescents. In addition, age and gender were shown to be important factors predicting nightmare increase, dream recall increase, report of an extraordinary dream, and pandemic-related dream content.

In general, we found an increased dream recall for 31% of the sample and an increase in nightmares for 18%. Nearly 15% of the participants who wrote down a dream reported dreams that dealt overtly with the pandemic. Although the methodology differs between studies, these percentages are comparable to the findings in adults (Iorio et al., 2020; Schredl and Bulkeley, 2020).

None of the objective stress factors linked to the pandemic (i.e., the death or illness of someone close to the student) showed a significant relationship to the outcomes in the regression analyses, whereas in adults the direct experience of grief and losses due to COVID-19 is correlated with dream content overtly related to the pandemic (Barrett, 2020; Schredl and Bulkeley, 2020). This discordance could be explained by the fact that very few of our participants reported having a loved one fall ill with COVID-19 (6.7%) or dying from it (1.5%). The adolescents of the present sample lived in countries or, in the case of the Italian students, in regions of a country that were not under a direct and brutal COVID siege, as opposed to the Italian adults examined by previous investigations (Iorio et al., 2020). Thus, stress might be linked to mandatory confinement rather than to actual health issues caused by the pandemic, as some recent studies suggested (Husky et al., 2020; Vicario-Merino and Muñoz-Agustin, 2020).

Participants who indicated negatively changed relationships with parents and experiencing feelings such as sadness and anger due to the lockdown reported an increase in nightmares; that is, the subjective stress experienced in waking life was directly related to negative changes in dreaming, a finding that is in line with the continuity hypothesis (Schredl, 2003). Likewise, those having worries about another lockdown showed more pandemic-related dream content. On the other hand, the result that a positively changed relationship with parents was associated with a decrease in nightmares highlights the importance of positive adolescent–parent relationships in contributing to the well-being of adolescents (Ben-Zur, 2003; Giannakopoulos et al., 2009). According to a study by Fioretti et al. (2020) on adolescents during the lockdown, the “re-discovery” of family is among the main positive traits perceived in times of COVID-19. Nightmares not only include anxiety, fear, and terror, but also (although more rarely) other dysphoric emotions such as anger and sadness (Cuddy and Belicki, 1992; Zadra et al., 2006). Thus, the link between negative feelings (e.g., anger and sadness) during the day and increased nightmares can be considered plausible.

To summarize, waking life emotions have a stronger relationship to dreaming (small to medium effect sizes for emotional variables such as sadness, anger, and worries) than the objective pandemic-related events. In other words, dreams reflect through images what we think of ourselves and others (Iannaco et al., 2015) and what is emotionally important to us (Schredl, 2018).

Our findings also suggest possible resilience factors, as spending time with creative activities during the lockdown was related to less pandemic-related dream content. Accordingly, students who reported spending time in a creative way were also writing down their dreams more often (Bone and Corlett, 1968; Fitch and Armitage, 1989; Brand et al., 2011).

Differences between the three countries were very small (effect sizes ranged from *d* = 0.094 to *d* = 0.138), with Italian participants more willing to write down a dream and more often reporting an increase in nightmares. However, these differences were no longer significant if the variables measuring the pandemic-related effects on the person were statistically controlled (regression analyses): that is, the higher percentages of increased nightmares and pandemic-related dreams in Italy are explained by the stronger emotional response to the pandemic in this country, due to the fact that during the first wave of the pandemic in spring 2020 Italy was severely hit, with a high death toll in its Northern regions. Nevertheless, the lack of substantial differences between the three European countries supports the idea that the effects of the pandemic on dreaming are not influenced by country-specific factors but by the subjective distress related to the pandemic; dreams in critical times seem to react primarily to individual emotional responses and relationships. As our sample was homogeneous from a sociological point of view (secondary school students, many of them in high school, belonging to a social class that had the means to overcome the lockdown emergency in relatively comfortable living and economic conditions), it would be interesting to include adolescents from different socio-economic backgrounds in a future survey. One would expect that adolescents who were less privileged socio-economically might report even stronger effects of the pandemic on their dream life.

Age was associated with all four of the dream variables. An increase in dream recall (and in nightmares, single-variable analyses) was more likely to be reported by older adolescents. This is consistent with other findings: whereas dream recall frequency decreases with advancing age in adults (Funkhouser et al., 1999; Guénole et al., 2010; Nielsen, 2012; Schredl and Göritz, 2015; Mangiaruga et al., 2018), an increase during adolescence was found (Nielsen, 2012). Unfortunately, we did not assess the dream recall frequency in our sample. Nevertheless, one might hypothesize that the dream life of older adolescents, as they recall their dreams more often, is more likely to show effects linked to the pandemic compared to younger schoolmates who recall their dreams quite rarely. In order to support this line of thinking, it would be necessary to include a dream recall frequency measure into future questionnaires.

Younger adolescents were more willing to write down an extraordinary dream than older ones. This finding is compatible with studies showing that adolescents' self-disclosure online and offline increases during early adolescence and that early adolescents use online self-disclosure to rehearse offline self-disclosure skills (Valkenburg et al., 2005, 2011). Older adolescents, albeit knowing that the questionnaire was strictly anonymous, might have been more sensitive to privacy issues and unwilling to report dreams that included sensitive topics, for instance sexuality or acts of aggression. Furthermore, studies have shown that older adolescents prefer to share dreams with close people and peers (Georgi et al., 2012; Olsen et al., 2013). This could explain why older adolescents may have had an overall higher dream recall but were less willing to report dreams explicitly in an online questionnaire.

Early adolescents' dreams also contained more overtly pandemic-related dream content than did the dreams of older students; this finding needs further investigation. Notably, early adolescents spend much of their time within the family compared to older ones (Larson et al., 1996; Larson and Verma, 1999). For this reason the younger participants in this study might have felt relatively protected from the pandemic, experiencing it as a vaguely imperiling condition rather than as a direct threat. In accordance with a contemporary psychodynamic theory on dreams (Fagioli, 2009), one might speculate that their dreams therefore adopted pandemic-related images as “metaphors,” i.e., means of expressing oneiric thoughts about themselves and their relational context, both in a negative sense (representing an interpersonal threat through pandemic-related items) and in a positive sense (representing personal achievement with dream scenarios like “recovering from COVID-19” or “finding a vaccine”).

Gender influenced all four of the dream variables: dream recall increase, nightmare increase, report of an extraordinary dream, and pandemic-related dream. Our findings confirmed that girls were more involved in dreaming. As found for females participants in previous studies (Schredl and Reinhard, 2008; Georgi et al., 2012; Settineri et al., 2019), girls showed an increase in dream recall during the lockdown. Regarding the increase in nightmares, our study again was consistent with the literature (Levin, 1994; Nielsen et al., 2000, 2006; Schredl and Reinhard, 2011) as a considerably higher number of female students reported an increase in nightmares during the lockdown period. However, no significant gender difference for dreams overtly related to COVID-19 was found in our sample, in contrast to previous research. Barrett (2020), Iorio et al. (2020), and Pesonen et al. (2020) reported higher scores for female adults, whereas we found boys to report more pandemic-related dream content, but only with exceedingly small effect sizes (*d* = 0.09); this relationship should be investigated further in future research.

### Strength and Limitations

The present sample was non-representative, e.g., with a relatively high proportion of female participants (69%). To control for this, regression analysis included gender as an additional factor, i.e., the reported associations between waking life parameters and dream variables are not affected by this sample characteristic.

Given the cross-sectional study design, the direction of the associations is difficult to interpret. The questions were aimed at the correlations between dreaming and the subjectively experienced effect of the lockdown on well-being in waking life, therefore we cannot exclude self-selection bias in our sample: for example, we might have adolescents in the sample that were strongly affected by the pandemic and wanted to share these effects with the researcher. However, one has to keep in mind that the study was not advertised as being dream related, so the bias regarding dreaming (e.g., an over-representation of high-dream recallers) should be minimal. A drawback here is that our questionnaire only included three dream-related questions. It would have been helpful if dream recall frequency, nightmare frequency, nightmare distress, and positive and negative emotions experienced in the dream had also been elicited. Moreover, the questionnaire was created under time pressure because of the ongoing pandemic. It is therefore not validated and the instruments used to measure emotional reactions during wakefulness (sadness, anger, and boredom) were not standardized. In addition, we asked our participants to write down an “extraordinary dream during this (pandemic) period,” hence the analytical findings on dream content cannot be compared to diary studies or recent dream studies that typically collect all dreams that the participants can remember (Domhoff, 1996). On the other hand, this approach allowed us to elicit the most striking dreams of the participants (i.e., the dreams that stuck out in their memory) and thus might be best suited to reflect the waking life stress related to the pandemic. We did not compute an inter-rater analysis, but recent research has shown that ratings of nominal scales, as with the present rating of presence or absence of pandemic-related themes, usually have very high inter-rater reliability indices (Schredl et al., 2004). As a key limitation, the study reports no information about sleep quality although the relationship between sleep and dream recall is well-known and the reported associations between waking life stress due to the pandemic and dreaming might be mediated by sleep parameters like decreased sleep quality. This would be an interesting question for future research. Nevertheless, our replication of previous findings of associations between waking life and dreaming suggest that our findings are valid. The strength of our study is our focus on adolescence, which in the literature has rarely been covered regarding dreams, especially in critical situations such as a pandemic. Furthermore, we focused on self-rated emotional and health data more than on the pathology.

## Conclusion

Our study indicated that reactions to the pandemic have a strong relationship to dreaming in adolescents, especially in those who experience emotional distress, such as anger or sadness, due to the pandemic. These relationships are in line with psychodynamic approaches to dreams (Fagioli, 1972) and the continuity hypothesis of dreaming (Schredl, 2003). The results were similar in Italy, Romania, and Croatia (after controlling for the emotional impact of the pandemic), indicating that the pandemic produces worldwide effects on dreams. The present findings encourage further studies on the inclusion of dreams in preventive programs for adolescents with high pandemic-related stress levels.

## Data Availability Statement

The raw data supporting the conclusions of this article will be made available by the authors, without undue reservation.

## Ethics Statement

This study was conducted in accordance with the Declaration of Helsinki and followed the privacy recommendations released by the Italian Ministry of Education, University and Research (MIUR, 2020). Written informed consent to participate in this study was provided by the participants' legal guardian/next of kin.

## Author Contributions

Material preparation, data collection, and analysis were performed by AG-G, IN-G, CI, MV, AB, CB, and MS. The first draft of the manuscript was written by AG-G, IN-G, MS, AH, and CI. All authors commented on previous versions of the manuscript, contributed to the conception, design of the study, read, and approved the final manuscript.

## Conflict of Interest

The authors declare that the research was conducted in the absence of any commercial or financial relationships that could be construed as a potential conflict of interest.
